# Identifying Factors Associated With Dropout During Prerandomization Run-in Period From an mHealth Physical Activity Education Study: The mPED Trial

**DOI:** 10.2196/mhealth.3928

**Published:** 2015-04-13

**Authors:** Yoshimi Fukuoka, Caryl Gay, William Haskell, Shoshana Arai, Eric Vittinghoff

**Affiliations:** ^1^Institute for Health & AgingDepartment of Social and Behavioral SciencesUniversity of California, San FranciscoSan Francisco, CAUnited States; ^2^Institute for Health & AgingDepartment of Family Health Care NursingUniversity of California, San FranciscoSan Francisco, CAUnited States; ^3^Stanford Prevention Research CenterStanford UniversityPalo Alto, CAUnited States; ^4^Institute for Health & AgingUniversity of California, San FranciscoSan Francisco, CAUnited States; ^5^Department of Epidemiology & BiostatisticsUniversity of California, San FranciscoSan Francisco, CAUnited States

**Keywords:** run-in period, eligibility, randomized controlled trial, pedometer, mobile phone, mHealth

## Abstract

**Background:**

The mobile phone-based physical activity education (mPED) trial is a randomized controlled trial (RCT) evaluating a mobile phone-delivered physical activity intervention for women. The study includes a run-in period to maximize the internal validity of the intervention trial, but little is known about factors related to successful run-in completion, and thus about potential threats to external validity.

**Objective:**

Objectives of this study are (1) to determine the timing of dropout during the run-in period, reasons for dropout, optimum run-in duration, and relevant run-in components, and (2) to identify predictors of failure to complete the run-in period.

**Methods:**

A total of 318 physically inactive women met preliminary eligibility criteria and were enrolled in the study between May 2011 and April 2014. A 3-week run-in period was required prior to randomization and included using a mobile phone app and wearing a pedometer. Cross-sectional analysis identified predictors of dropout.

**Results:**

Out of 318 participants, 108 (34.0%) dropped out prior to randomization, with poor adherence using the study equipment being the most common reason. Median failure time was 17 days into the run-in period. In univariate analyses, nonrandomized participants were younger, had lower income, were less likely to drive regularly, were less likely to have used a pedometer prior to the study, were generally less healthy, had less self-efficacy for physical activity, and reported more depressive symptoms than randomized participants. In multivariate competing risks models, not driving regularly in the past month and not having used a pedometer prior to the study were significantly associated with failure to be randomized (*P*=.04 and .006, respectively), controlling for age, race/ethnicity, education, shift work, and use of a study-provided mobile phone.

**Conclusions:**

Regular driving and past pedometer use were associated with reduced dropout during the prerandomization run-in period. Understanding these characteristics is important for identifying higher-risk participants, and implementing additional help strategies may be useful for reducing dropout.

**Trial Registration:**

ClinicalTrials.gov NCT01280812; https://clinicaltrials.gov/ct2/show/NCT01280812 (Archived by WebCite at http://www.webcitation.org/6XFC5wvrP).

## Introduction

Given the exponential growth of mobile phone use—both basic- and advanced-feature mobile phones—across all age groups [1], mobile health technology has become a popular way to deliver physical activity interventions and monitor physical activity. Applying mobile phone technologies to a randomized controlled trial (RCT) has great potential to improve measurement and intervention methodologies. However, several important methodological questions, such as the value and limitations of run-in procedures for mobile technology-based RCTs, have not been adequately addressed [2], particularly for physical activity interventions.

An RCT is the gold standard for examining intervention efficacy and effectiveness. RCTs tend to focus on internal validity at the expense of external validity, and thus, study findings can have limited external validity (ie, generalizability) [3]. A run-in procedure has been proposed for RCTs [4] to minimize the challenges of attrition and nonadherence to the intervention being evaluated, which can be significant threats to trials’ internal validity [4]. Several RCTs have used this design [5,6]. Using a run-in period as part of mobile app-based physical activity intervention trials allows researchers to screen out ineligible (eg, already active) or noncompliant (eg, low adherence to app use) research participants prior to randomization, and thereby improve the internal validity of these RCTs. Run-in periods can be especially useful with technology-based interventions, since individuals often adopt and discontinue technology use at different speeds [4].

The mobile phone-based physical activity education (mPED) study [7] is an RCT with a run-in procedure and is designed to evaluate the efficacy of a mobile app-delivered physical activity intervention—for both basic- and advanced-feature mobile phones—for physically inactive women. An overall goal of this paper was to describe the process of selecting mPED participants prior to randomization and their characteristics. Generally, factors affecting run-in attrition include environmental factors (ie, physical and social environment, such as social support and program location), program factors (eg, design, recruitment processes, and eligibility criteria), and person-based factors (eg, demographics and beliefs about exercise) [8]. Understanding such characteristics among the physically inactive women who were randomized and those who were not is a critical part of evaluating the mPED study’s external validity, and could also yield useful information for guiding the implementation and possible dissemination of this mobile phone-delivered physical activity intervention. In addition, evaluating the timing and patterns of ineligibility can inform the design of run-in periods in future RCTs by determining optimum duration and relevant components.

Thus, the aims of this study were to (1) describe the timing of, and reasons for, nonrandomization in the mPED trial and (2) identify predictors of failure to complete the run-in period.

## Methods

### Study Design

The mPED study is an RCT that included a preliminary telephone screening call, a screening/baseline study visit, and a 3-week run-in period to determine participants’ eligibility for randomization. In this paper, mPED data from this prerandomization phase of the study were analyzed to compare the characteristics of randomized versus nonrandomized participants and to evaluate the timing of ineligibility during the 3-week run-in period. The study protocol was approved by the University of California, San Francisco Committee on Human Research and the mPED Data and Safety Monitoring Board. The study protocol was published in 2011 [7]. All potential participants received a copy of the informed consent form electronically or by mail after completion of telephone screening and were asked to review it before the screening/baseline visit. All participants provided written consent prior to study enrollment. This RCT was registered at ClinicalTrials.gov (NCT01280812).

### Subject Recruitment

Physically inactive women were recruited from the San Francisco Bay Area from May 2011 to April 2014. With the aim of recruiting a diverse and representative sample, four broad types of subject recruitment strategies were used: (1) media advertising (eg, newspaper, radio, Craigslist and Facebook ads, commercial email distribution lists, and study, clinic, and ClinicalTrials.gov websites), (2) posting fliers in the community (eg, stores, bus stops, medical and dental clinics, community centers, university campuses, and churches), (3) random mailing of the study announcement to women aged 25 to 69 who live in San Francisco, and (4) referral from friends, family members, health care providers, or others contacts.

### Inclusion and Exclusion Criteria

Preliminary inclusion criteria were assessed during the telephone screening and included the following: (1) female, aged 25 to 69 years, (2) body mass index (BMI) of 18.5-43.0 kg/m^2^, (3) physically inactive lifestyle as indicated by a Stanford Brief Activity Survey [9] score indicating inactivity or light activity during leisure time and at work, if employed, (4) intent to become physically active, (5) willingness to use the pedometer and intervention app every day for 9 months, (6) access to a home telephone or mobile phone, and (7) ability to speak and read English. Preliminary exclusion criteria included the following: (1) known medical conditions or physical problems that require special attention in an exercise program, (2) planning an international trip during the next 4 months, which could interfere with daily server uploads of mobile phone data, (3) pregnant/gave birth during the past 6 months, (4) severe hearing or speech problem, (5) history of an eating disorder, (6) current substance abuse, (7) current participation in lifestyle modification programs or research studies that may confound study results, and (8) history of bariatric surgery or plans for bariatric surgery in the next 12 months. Women who had never used a mobile phone or were not current mobile phone users were not excluded.

Additional inclusion criteria were assessed at the screening/baseline visit or during/after the run-in period and included the following: (1) a physical exam confirming BMI and medical eligibility information obtained during the initial telephone screening (eg, height, weight, resting blood pressure), (2) a fasting blood test confirming medical eligibility, (3) a baseline average of <8500 daily steps measured during the run-in period, and (4) at least 80% adherence to all run-in activities (described below). Participants were also assessed using the Mini-Cog test [10,11] and were excluded if there was evidence of mild cognitive impairment.

### Telephone Screening

During the initial screening call, a trained study staff member screened potential participants for preliminary eligibility. Potential participants who met preliminary eligibility criteria were invited to attend a screening/baseline visit and were sent the study consent form, public transportation and parking information, directions to the research office, and a list of study requirements, which included a picture of the pedometer they would be asked to wear.

### Screening/Baseline Visit

The screening/baseline visit was scheduled approximately 1 week after completion of the telephone screening. The primary goal of the screening/baseline visit was to further determine the participant’s eligibility. Once written informed consent was obtained, participants were screened for mild cognitive impairment using the Mini-Cog test and were asked to complete baseline questionnaires (described in the Measures section below) and a physical exam. Eligible participants were issued a mobile phone and a pedometer (described below)—training was provided to ensure participants could successfully use both devices. The mean training time for the use of the mobile phone and pedometer was 14.1 (SD 6.3) minutes.

### Run-in Period

#### Overview

The run-in period lasted approximately 3 weeks. During that time, participants were asked to wear the pedometer, use the mobile phone app, and have a fasting blood test.

#### Pedometer

The Omron Active Style Pro HJA-350IT with triaxial accelerometer was selected for this trial because it has well-established reliability and validity, has been used in similar studies, and records 150 days of activity data. Its dimensions are 74x46x34 mm (width/height/depth) including the clip, and it weighs 60 grams (2.1 oz), including batteries. The pedometer was set to record and store physical activity (eg, steps), but not to display the step counts—only date and time were displayed to limit reactivity. Participants were asked to wear the pedometer all day, except when showering/bathing, swimming, or sleeping, from the time they got up in the morning until they went to bed at night for the duration of the run-in period. Participants were asked to wear the pedometer on their waist, aligned with their dominant knee.

#### Mobile Phone App

A run-in mobile phone app was created specifically for this phase of the study—it was designed to mimic the intervention app without any content to encourage or support increasing physical activity. A Java 2 Platform Micro Edition (J2ME) app for basic-feature mobile phones was initially developed for the study, and later iPhone (iOS) and Android apps were developed. None of the delivery platforms (ie, J2ME, iOS, and Android) required Internet connectivity. The run-in app sent daily messages unrelated to physical activity throughout the run-in period (eg, “Did you eat breakfast today?”), and participants were instructed to respond to each message. In addition, participants were instructed to enter an estimate of their daily step count into the app’s daily activity diary every day of the run-in period. Adherence to these instructions was monitored remotely, and participants with low adherence were contacted by project staff to troubleshoot problems and alert them that they were at risk for not meeting the run-in criteria. The app could be installed on a participant’s personal phone if they had a compatible mobile phone, and the study reimbursed the cost of upgrading their text and data plans to cover study-related use. Alternatively, participants were provided with a mobile phone for the purpose of the study. Study-issued mobile phones had unlimited data and text messaging and 70 minutes of voice calls per month, and no restrictions on personal use.

#### Fasting Blood Test

Participants were also asked to complete a fasting blood test in a clinical research lab during the first week of the run-in period. Blood samples were assayed to obtain a lipid profile, fasting plasma glucose, and hemoglobin A1c (HbA1c) values.

The run-in period had three purposes: (1) to determine a baseline average number of daily steps, (2) to determine the participant’s level of adherence to the study procedures, and (3) to obtain baseline fasting blood test results to evaluate the potential risks or benefits of participating in the study. If a participant had five or more cardiovascular risk factors, research staff, with the participant’s permission, sent a study enrollment notification letter to their health care provider.

### Randomization Visit and Adherence Indicators

At the conclusion of the run-in period, participants were scheduled for a randomization visit. At this visit, the pedometer data were downloaded and reviewed to ensure that the participant met the criterion for a daily average of <8500 steps across the run-in period. Adherence indicators for both the pedometer and mobile phone app were also reviewed to ensure 80% adherence to each of the following criteria: (1) pedometer-wearing time of at least 8 hours per day, (2) responding to the app’s daily messages, and (3) using the app’s daily activity diary. In addition to consistently using the pedometer and mobile phone app throughout the run-in period, participants were required to successfully complete the fasting blood test to be eligible for randomization.

### Participant Payments

Participants ineligible to start the run-in were paid US $10 in cash at the screening/baseline visit. Participants who completed the run-in, but were not randomized were paid US $20 in cash at the randomization visit. Randomized participants received a US $40 check when they completed the 3-month study visit. Parking was provided at the research office, and participants were reimbursed for parking expenses for the blood draw. Those who took public transportation did not receive specific reimbursement for their transportation.

### Measures

#### Overview

Sociodemographics, lifestyle and health characteristics, and past digital technology use were assessed during the telephone screening or screening/baseline visit and are summarized in Table 1. Total television and computer usage (hours per week) was assessed using a questionnaire developed by the research team based on a thorough review of the literature. In addition, the following four standardized and validated questionnaires were administered at the screening/baseline visit.

#### Self-Efficacy for Physical Activity Scale

A 6-item modified version of the original 5-item Self-Efficacy for Physical Activity Scale [12] was used to assess confidence in one’s ability to exercise, an important determinant of the stages of change for exercise behavior. The scale assesses one’s perceived ability to exercise despite common challenges (ie, bad weather, limited time, feeling tired, bad mood, or being on vacation). Based on a pilot study [13], the scale was modified to include a sixth item assessing one’s ability to exercise during times of stress. Total scores can range from 6 to 30, with higher scores indicating greater self-efficacy for physical activity.

#### Social Support for Exercise Survey

The Social Support for Exercise Survey consists of 13 items assessing the level of perceived support from family and friends for behavior changes related to exercise [14]. Each item is scored separately for family and friends, and scores can range from 13 to 65, with higher scores indicating greater support.

#### Barriers to Being Active Quiz

The Barriers to Being Active Quiz consists of 21 items assessing seven types of barriers to physical activity: lack of time, lack of social influence, lack of energy, lack of willpower, fear of injury, lack of skill, and lack of resources [15]. Scores can range from 0 to 63, with higher scores indicating more barriers to physical activity.

#### Center for Epidemiological Studies Depression Scale

The Center for Epidemiological Studies Depression Scale (CES-D) is a 20-item questionnaire widely used for assessing symptoms of depression [16]. Scores can range from 0 to 60, with higher scores indicating more depressive symptoms.

#### Definition of Time to Drop Out

The primary outcome was time to drop out, defined as the number of days between the screening/baseline visit and the earliest indicator of run-in failure. This outcome was measured using the following: (1) the date a participant called or emailed to withdraw from the study, or (2) the date a participant entirely stopped wearing the pedometer or using the mobile phone app. For participants who completed the run-in period, the time to drop out was defined as the number of days between the screening/baseline visit and the randomization visit.

### Statistical Analysis

Sample size was based on the primary outcomes of the RCT [7]. Descriptive statistics were used to summarize participant characteristics. Comparisons of randomized and nonrandomized participants were conducted using chi-square tests or independent sample *t* tests, as appropriate. Fine-Gray competing risk models [17] were used to estimate adjusted covariate effects on dropout during the run-in period, accounting for successful randomization as a competing risk. All variables in Table 1 were evaluated as potential predictors. The final adjusted model was obtained using forward selection with an entry criterion of *P*<.10 except that age, race/ethnicity, education, shift work, and use of a study-provided mobile phone were included and retained by default for face validity. Cumulative incidence of dropout was estimated by the baseline cumulative incidence function of a simple Fine-Gray model with no covariates. Analyses were conducted using Stata version 13.1 (Stata Corp, College Station, TX).

## Results

### Subject Enrollment and Dropout

**Figure 1:**
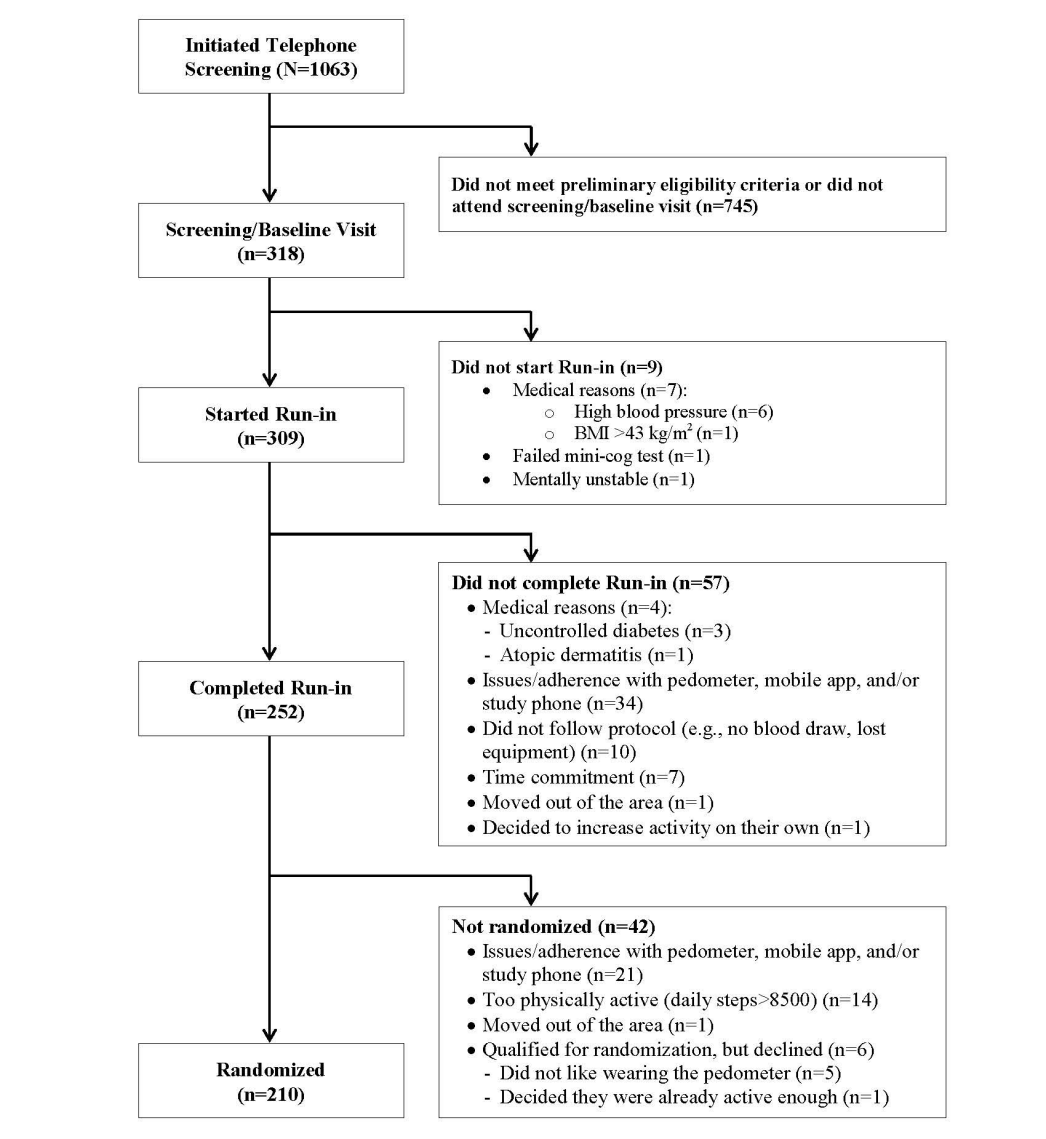
Flow diagram of the mPED run-in period.

A total of 1063 potential participants were screened by telephone for initial eligibility. Of these, 745 (70.08%) did not meet the initial eligibility criteria or did not attend the screening/baseline visit, and the remaining 318 (29.92%) women were initially eligible, enrolled in the study, and completed the screening/baseline visit. Of the 318 participants enrolled in the study, 210 (66.0%) successfully completed the run-in period and were randomized to one of the three groups, and the remaining 108 (34.0%) were not randomized for various reasons, as listed in Figure 1. The most common reasons for nonrandomization were issues related to the pedometer, study phone, and/or mobile app (55/108, 50.9%). Among the 108 participants who were not randomized, the median failure time was 17 days into the run-in period. Issues/adherence problems with the pedometer included not wanting to wear it because it was bulky, uncomfortable, or difficult to wear and failing to wear it 8 hours per day on at least 80% of the run-in days. Issues/adherence problems with the mobile app included not liking aspects of it (eg, the timing of the messages and diary), not understanding the instructions, not meeting the 80% adherence rate, as well as mobile app glitches. Issues with the phone included not liking the study-provided mobile phone and finding it difficult to use.

### Comparisons of Randomized and Nonrandomized Participants

Table 1 summarizes univariate comparisons between the 210 randomized participants and 108 nonrandomized participants. Compared to randomized participants, nonrandomized participants were younger, had lower income, had a lower self-rating of general health, reported less self-efficacy for physical activity and more depressive symptoms, were less likely to drive on a regular basis, and were less likely to have used a pedometer prior to the study. Downloading the mPED app to one’s own phone was not associated with randomization or age (*P*=.33). Although not statistically significant, nonrandomized participants were slightly less likely to have participated in a weight-loss or diet program and had slightly higher scores on the Barriers to Being Active scale.

**Table 1:** Univariate comparisons of randomized and nonrandomized participants (n=318).

Measure				Nonrandomized participants (n=108), n (%) or mean (SD)	Randomized participants (n=210), n (%) or mean (SD)	*P*
**Sociodemographics and lifestyle characteristics**						
	Age (years), mean (SD)			49.0 (12.4)	52.4 (11.1)	*.01* ^a^
	**Race/ethnicity, n (%)**					.69
			Native Hawaiian/Pacific Islander	1 (0.9)	0 (0.0)	
			Black/African American	9 (8.3)	17 (8.1)	
			Hispanic/Latino	7 (6.5)	13 (6.2)	
			Asian	24 (22.2)	41 (19.5)	
			White (non-Hispanic)	60 (55.6)	119 (56.7)	
			More than one race/ethnicity	7 (6.5)	20 (9.5)	
	**Education, n (%)**					.81
		Completed high school or some college		25 (23.1)	52 (24.8)	
		Completed college		42 (38.9)	86 (41.0)	
		Graduate school		41 (38.0)	72 (34.3)	
	**Household income (annual), n (%)**					*.03*
		≤ US $40,000		30 (27.8)	32 (15.2)	
		US $40,001 to $75,000		27 (25.0)	50 (23.8)	
		> US $75,000		42 (38.9)	111 (52.9)	
		Don’t know/declined to state		9 (8.3)	17 (8.1)	
	**Marital status, n (%)**					.82
		Never married		35 (32.4)	64 (30.5)	
		Currently married/cohabitating		51 (47.2)	107 (51.0)	
		Divorced/widowed		22 (20.4)	39 (18.6)	
	**Employment and shift work, n (%)**					.66
		Full- or part-time job with no shift work		52 (48.1)	108 (51.4)	
		Full- or part-time job with shift work		23 (21.3)	48 (22.9)	
		No paid employment		33 (30.6)	54 (25.7)	
	Has a dog, n (%)			22 (20.4)	44 (21.0)	.90
	Smoked a cigarette during the past 7 days, n (%)			3 (2.8)	4 (1.9)	.62
	Drove at least once a week during the last month, n (%)			80 (74.1)	176 (83.8)	*.04*
	Prior weight-loss or diet program participation, n (%)			56 (51.9)	132 (62.9)	.06
	Used a pedometer prior to the study, n (%)			38 (35.2)	109 (51.9)	*.005*
**Technology use, n (%)**						
	Used a mobile phone at least once a week during the last month			104 (96.3)	200 (95.2)	.66
	Used a computer or accessed the Internet at least once a week during the last month			106 (98.1)	208 (99.0)	.49
	Owns advanced mobile phone			70 (64.8)	125 (59.5)	.36
	Subscribed to text messaging plan (n=316)^b^			88/108 (81.5)	158/208 (76.0)	.66
	Used Facebook during the last month (n=303)^b^			64/98 (65.3)	140/205 (68.3)	.60
	Used their own phone for the study (n=313)^c^			36/103 (35.0)	70/210 (33.3)	.78
	**Type of mobile phone used during the study (n=313)** ^c^					.31
		Motorola		12/103 (11.7)	16/210 (7.6)	
		Pantech		31/103 (30.1)	51/210 (24.3)	
		iPhone		59/103 (57.3)	142/210 (67.6)	
		Android		1/103 (1.0)	1/210 (0.5)	
**Health characteristics**						
	Overall rating of general health (scale 1-7), mean (SD)			4.74 (1.14)	5.00 (1.05)	*.01*
	Measured body mass index (kg/m^2^), mean (SD)			29.2 (6.1)	29.9 (6.2)	.34
	Self-reported high blood pressure, n (%)			28 (25.9)	52 (24.8)	.98
	Self-reported high cholesterol, n (%)			26 (24.1)	71 (33.8)	.16
	Self-reported prediabetes or type 2 diabetes, n (%)			7 (6.5)	16 (7.6)	.71
**Self-report questionnaires, mean (SD)**						
	Self-Efficacy for Physical Activity Scale			18.0 (5.1)	19.2 (4.6)	*.04*
	**Social Support for Exercise Survey**					
		Family		31.8 (9.7)	32.1 (9.7)	.81
		Friend		32.2 (7.8)	31.5 (8.4)	.43
	Barriers to Being Active: total score			25.5 (9.7)	23.4 (10.1)	.08
	Depressive symptoms (CES-D^d^)			12.1 (9.6)	9.7 (7.6)	*.02*
Total television and computer usage (hours per week), mean (SD)				28.5 (20.2)	27.5 (18.5)	.67
**Participant recruitment strategies, n (%)**						.67
	Media advertising			29 (26.9)	66 (31.4)	
	Posting fliers in the community			36 (33.3)	62 (29.5)	
	Selective mailing			31 (28.7)	53 (25.2)	
	Referral from friends, family members, health care providers, or other contacts			12 (11.1)	29 (13.8)	

^a^ *P* values <.05 appear in italics.

^b^Missing data.

^c^Study mobile phones were not issued to 5 excluded participants at the start of the run-in period.

^d^Center for Epidemiological Studies Depression Scale (CES-D).

### Predictors of Time to Failure During the Run-in Period

Table 2 summarizes the adjusted subdistribution hazard ratios (SHRs) for covariate effects on failure during the run-in period, accounting for the competing risk of successful randomization. In models adjusting for all variables in Table 2, not driving and not having used a pedometer before were the only predictors that remained statistically significant (*P*<.05). Sensitivity analyses excluding the 9 participants ineligible to start the run-in period indicated no meaningful change in the results reported in Table 2.

**Table 2:** Multivariate subdistribution hazard models predicting time to failure during the run-in period (n=313).

Predictors			SHR^a^ (95% CI)	*P*
**Face validity predictors**				
	Age		0.98 (0.97-1.00)	.09
	**Race/ethnicity**			
		Other^b^ (reference)	1	
		White (non-Hispanic)	1.12 (0.74-1.70)	.60
	**Education**			
		Completed high school (reference)	1	
		Completed college	1.14 (0.66-1.96)	.65
		Completed graduate school	1.58 (0.91-2.76)	.11
	**Employment status**			
		Employed, day shift (reference)	1	
		Employed with shift work	1.23 (0.72-2.12)	.45
		Unemployed	1.52 (0.97-2.37)	.07
	Used their own phone for the study		1.05 (0.70-1.59)	.81
**Other predictors**				
	Drove at least once a week in the past month		0.61 (0.38-0.98)	*.04* ^c^
	Used a pedometer prior to the study		0.56 (0.37-0.85)	*.006*
	Self-efficacy for physical activity		0.96 (0.91-1.00)	.05

^a^Subdistribution hazard ratio (SHR).

^b^Includes Native Hawaiian/Pacific Islander, black/African American, Hispanic/Latino, Asian, and more than one race/ethnicity.

^c^ *P* values <.05 appear in italics.

## Discussion

### Principal Findings

To our knowledge, this study represents the first report to examine participant characteristics in relation to the timing of dropout during the run-in period of an intervention trial utilizing both mobile phone and pedometer technology. Overall, 34.0% (108/318) of the women who successfully completed the screening/baseline visit were not randomized, and the median time from the screening/baseline visit to dropout was 17 days. Equipment issues were the most common reasons for not being randomized, specifically trouble using the study phone or mobile phone app and refusal or failure to consistently wear the pedometer. Furthermore, women who had never used a pedometer prior to the trial were more likely not to be randomized, compared to those with a prior history of pedometer use. This finding could be explained by the study requirement of wearing a large pedometer all day throughout the 3-week run-in period. Wearing a relatively large pedometer every day for 9 months can be a considerable challenge for some women, particularly those who wear dresses or do not like the appearance of a large device clipped to their clothing. Although a picture of the pedometer was sent to participants immediately after the telephone screening, those who had never used a pedometer before may not have realized what they looked like or what it would be like to wear one every day. This finding highlights the importance of assessing past pedometer usage and helping potential participants understand all study requirements before the screening/baseline visit.

Women who drove regularly in the month prior to study enrollment were more likely to be randomized compared to those who did not. This finding could be explained by the fact that the study provided parking stickers to participants who drove to the study office, but no reimbursement was provided for public transportation. As reported in other studies [3,18], providing transportation/parking to study participants appeared to be critical to participant retention in this physical activity trial. In addition, receiving a separate reimbursement for travel may increase study retention, even when the total amount is the same as when provided in a single payment [19]. Providing door-to-door service or no-cost transportation appears to be important, particularly for nonwhite women. Basing studies in the communities they aim to serve may also reduce transportation barriers for women who do not drive regularly. The participant retention rate in relation to the research costs associated with these services needs to be evaluated when designing a study.

In contrast, neither older age nor use of one’s own mobile phone for the study had a significant effect on attrition during the run-in period, even though a small proportion 14/318 (4.4%) of enrolled women did not regularly use a mobile phone. Researchers often assume that older participants and subjects who do not download the study app onto their own mobile phone will have high attrition rates, but neither of these assumptions was supported by the findings of this study. Other recent papers on weight-loss interventions also concluded that younger women were more likely to drop out from weight-loss clinical trials compared to older women [20,21]. In this study, a similar trend was observed. We believe that providing a brief mPED app training session, as well as the simple app design and use of the app prior to the run-in period, helped older women to start and continue the run-in period.

Mobile phone technologies are evolving at an exponential rate to better meet the needs of consumers, and these technological improvements can also be incorporated into clinical trials to improve the experience of study participants. However, rapid adaptation to these changing technologies in the middle of a clinical trial can be challenging, if not impossible. Despite this challenge, three versions of the mPED app were developed and used over the 3-year study period. Yet, only 106/313 (33.9%) of the participants used the mPED app on their own mobile phone during the run-in period, while the remaining participants used an mPED study mobile phone.

### Strengths and Limitations

The findings from this study have methodological implications for researchers who design clinical trials involving mobile phone and pedometer technologies. The inclusion of subjects with diverse characteristics and the extensive technology information collected allowed us to explore these factors in relation to attrition during the run-in period. However, several study limitations need to be acknowledged. Only women aged 25 to 69 years were included, and thus, the findings may not generalize to men or children. Also, the study provided free parking, but did not provide a separate reimbursement for public transportation costs, which may have decreased retention, particularly among lower-income participants who are less likely to drive. In addition, San Francisco Bay Area’s extensive public transportation network makes it possible to access the research office without a car, and thus, the effect of driving status on attrition might be different in areas with more limited public transportation. Finally, the study was not able to distinguish between the different types of nonadherence because participants who struggled with one form of technology often gave up on the others as well. Future studies should develop more rigorous ways of independently assessing adherence to different technologies.

### Conclusions

In conclusion, regular driving and pedometer use prior to the study were associated with reduced dropout from the mPED trial’s prerandomization run-in period. Understanding these characteristics is important when interpreting results of the mPED trial or when designing a similar physical activity trial in the future.

## Abbreviations

BMI: body mass index
CES-D: Center for Epidemiological Studies Depression Scale
HbA1c: hemoglobin A1c
J2ME: Java 2 Platform Micro Edition
mPED: mobile phone-based physical activity education
RCT: randomized controlled trial
SHR: subdistribution hazard ratio
